# Computational Simulations of Provisional Stenting of a Diseased Coronary Artery Bifurcation Model

**DOI:** 10.1038/s41598-020-66777-1

**Published:** 2020-06-15

**Authors:** Henry Y. Chen, Yiannis S. Chatzizisis, Yves Louvard, Ghassan S. Kassab

**Affiliations:** 1 California Medical Innovations Institute, San Diego, CA United States; 2 Cardiovascular Biology and Biomechanics Laboratory, University of Nebraska Medical Center, Omaha, NE United States; 3 Institut Cardiovasculaire Paris Sud, Moassy, France

**Keywords:** Biomedical engineering, Applied mathematics

## Abstract

Although stenting of non-branched arterial segments has acceptable clinical outcomes, in-stent restenosis (ISR) and stent thrombosis remain clinically significant issues for vascular bifurcations (15–28% restenosis). Local fluid and solid stresses appear to play an important role in restenosis and thrombosis. The combined role of wall shear stress (WSS) and circumferential wall stresses (CWS) is unclear in the case of stenting at vascular bifurcations. Using numerical simulations, we computed the fluid shear, solid stresses and the stress ratio at the the bifurcation region. Stenting of main vessel increased the maximum CWS in the the side branch (SB), resulting in a nearly two-fold increase of stress ratio in the SB compared to the MB (5.1 × 10^5^ vs. 9.2 × 10^5^). The existence of plaque decreased WSS and increased CWS near the carina, increasing the stress ratio at the SB. The changes of stress ratio were highly consistent with clinical data on bifurcation stenting. Fluid dynamics and solids mechanics should be considered in planning of stenting for a specific bifurcation, as their combined biomechanical effect may play an important role in stent restenosis and thrombosis.

## Introduction

Although stenting of non-branched vessels has shown acceptable safety and efficacy, bifurcation stenting is associated with higher rates of adverse events and remains a challenge among the interventional cardiology community. Risks of ISR and late stent thrombosis are elevated for bifurcation lesions, resulting in higher complication rates and more frequent re-intervention^1–4^. Restenosis in vessels treated by drug eluting stent (DES) is still elevated in vascular bifurcations. DES does not resolve the flow disturbances or intramural wall stress alterations which can promote ISR^5,6^. Clinical data reported that SB has much higher re-stenosis rate than that of the main vessel in provisional stenting^7^.

The biomechanical stresses (wall shear stress, WSS; circumferential wall stress, CWS) are known to affect biological responses in blood vessels^8,9^. High solid CWS can stimulate smooth muscle cell proliferation, leading to hyperplasia. Additionally, low fluid WSS enhances inflammatory processes and promote thrombosis^10–12^. Computational simulation is a very useful tool to evaluate the local biomechanical stresses^5,6^. Computational fluid dynamics (CFD) has been known as a useful tool to evaluate the endothelial shear stress in blood vessels^6,9^. Computational studies also allow enable virtual simulations of stenting procedures rather than time-consuming experimental testing.

The objective of this study is to understand the fluid and solid mechanical disturbances caused by provisional stenting in bifurcations with the presence of plaque structure. These local biomechanical perturbations may affect ISR as higher stress ratio correlates with higher restenosis rates. We developed computational models of bifurcation stenting and simulations with and without the presence of plaque. We then compared the simulations results with relevant clinical data to study the potential relationship.

## Methods

**Figure 1:**
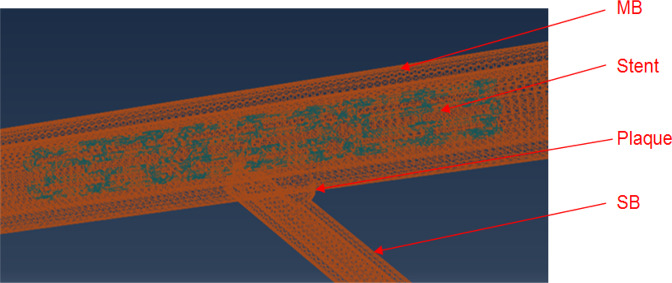
The structured and refined meshes of bifurcation with plaque and stent. The relative positions of stent and the bifurcation (MB and SB) are shown.

Computational models of stent, plaque and vascular bifurcation were created and then meshed and solved (Fig. 1). The configuration of stent and plaque are shown in Fig. 1.

### Governing equations

The governing equations for blood flow are the Navier-Stokes and Continuity equations; i.e., conservation of momentum and mass:

1 $$\frac{\partial \overrightarrow{V}}{\partial t}+\overrightarrow{V}\cdot \overrightarrow{\nabla }\overrightarrow{V}+\frac{{\overrightarrow{\nabla }}_{p}}{\rho }-2\frac{\eta }{\rho }\overrightarrow{\nabla }\cdot D=\overrightarrow{0}$$

2 $$\overrightarrow{\nabla }\cdot \overrightarrow{V}=0$$

where *V* is fluid velocity, *P* is fluid pressure, *ρ* is fluid mass density, *η* is fluid dynamic viscosity, $\overrightarrow{\nabla }$ is the gradient operator. *D* is the fluid rate of deformation tensor.

The governing equations for the solids were the Momentum and Equilibrium equations; i.e., Newton’s laws of Mechanics:

3 $$\begin{array}{ccccc}\rho {a}_{i}-{\sigma }_{ij,j}-\rho {f}_{i} & = & 0 & in & {}^{s}\Omega (t)\end{array}$$

4 $$\begin{array}{ccccc}{\sigma }_{ij}{n}_{j}-{t}_{i} & = & 0 & on & {}^{s}\varGamma (t)\end{array}$$

where *a*_*i*_ is acceleration, *f*_*i*_ is force per unit mass, ^*s*^Ω(*t*) is the vessel domain at time t, *n*_j_ is normal vector, *t*_*i*_ is surface traction vector, and σ_*ij*_ is stress.

The stent struts interact with the vessel wall by the Augmented Lagrangian contact algorithm. To model the interactions, contact surfaces were defined. The vessel wall was constrained in the proximal and distal sections to prevent free body movement.

### Flow simulations

The blood was modeled with flow based on human coronary artery velocity measurements applied at the inlet of artery^13,14^. For the outlet, a traction free boundary condition was imposed^5^. The flow was assumed to be non-turbulent. To evaluate the effects of both the solid and fluid mechanics, we introduced a stress ratio as Solid CWS/Fluid WSS. This stress ratio was previously validated for investigation of mechanical disturbances within blood vessels^5^.

### Ethical approval

The study was approved by the ethical committee of California Medical Innovations Institute.

## Results

The Reynolds numbers in the current study was an order of magnitude smaller than the Reynolds number for transition to turbulence (>2,300). Therefore, the current non-turbulent models were appropariate.

**Figure 2:**
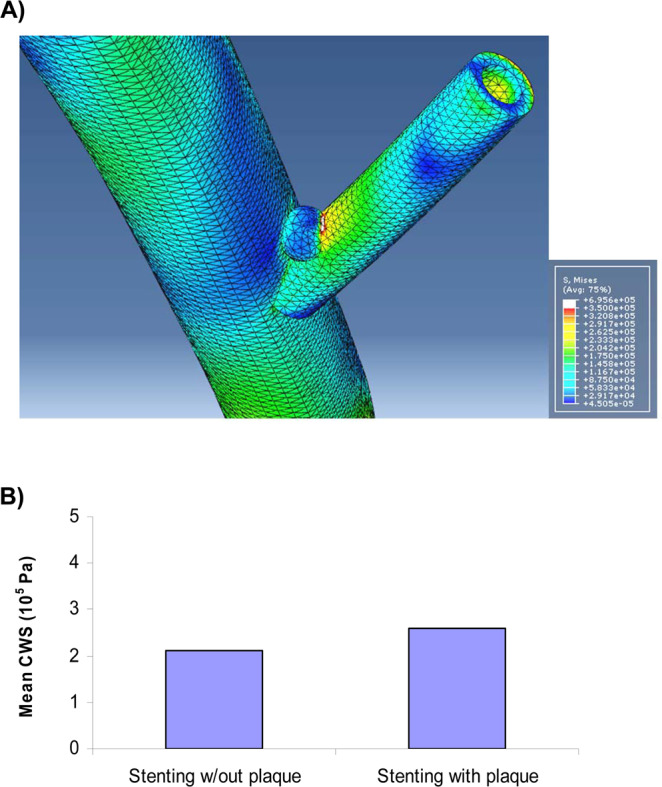
(**A**) For provisional stenting with bifurcation plaque, the stress distribution shows stress concentration at the SB, near the carina. The plaque was non stenotic. (**B**) Stenting of bifurcation with plaque resulted in higher stresses than that of without plaque.

**Figure 3:**
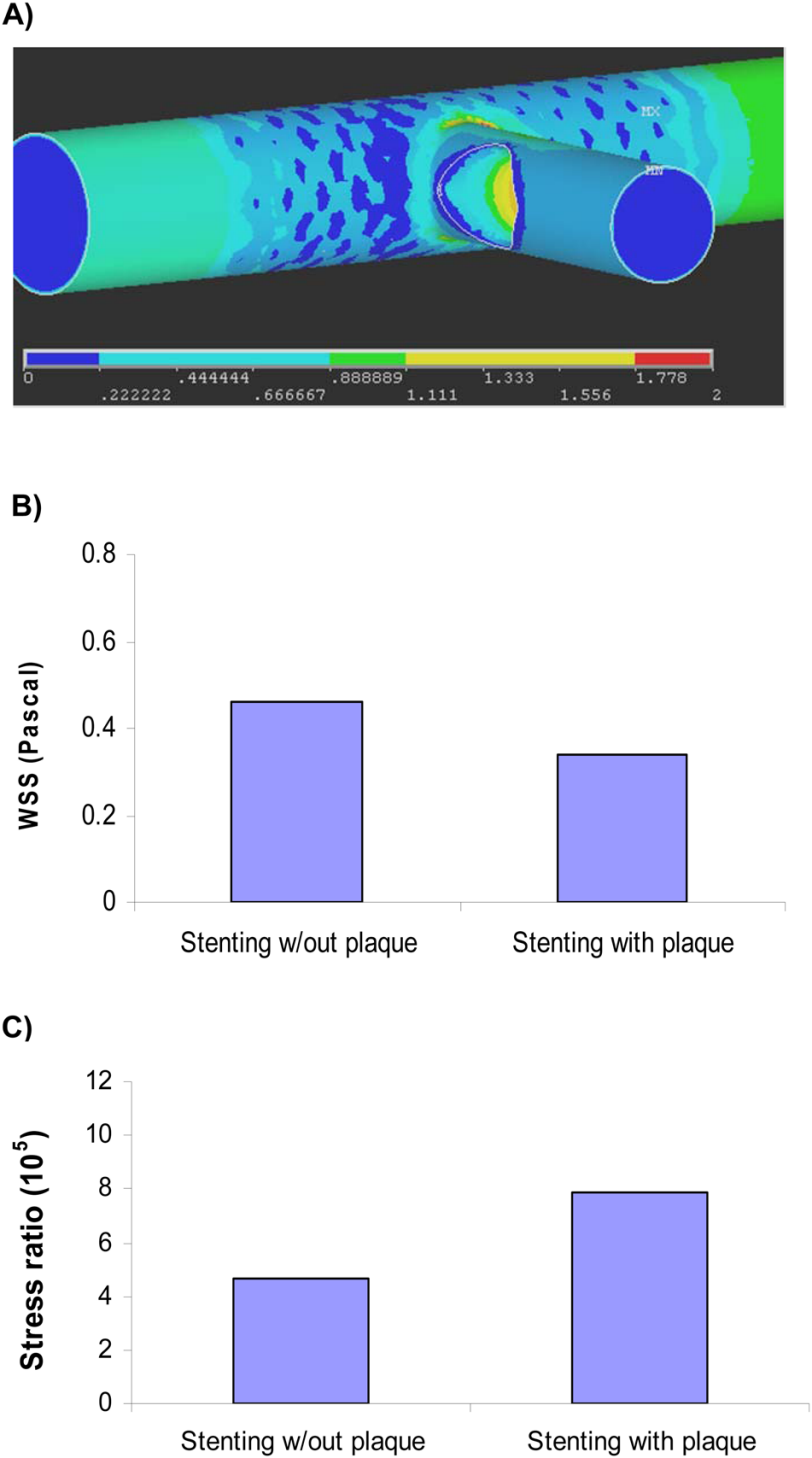
(**A**) The fluid WSS distribution in stenting of bifurcation with plaque. The areas around the carina had lower WSS. (**B**) Stenting of bifurcation with plaque resulted in lower WSS than that of without plaque. (**C**) The simulations found that stenting of bifurcation with plaque resulted in higher stress ratio than that of without plaque (4.7 × 10^5^ vs. 7.9 × 10^5^).

For provisional stenting with bifurcation plaque, the stress distribution shows stress concentration at the SB, at interface with plaque near the carina (Fig. 2A). Stenting of bifurcation with plaque resulted in higher mean stresses than that of without plaque (270 KPa vs. 220 KPa) (Fig. 2B). The fluid WSS distribution in stenting of bifurcation with plaque is shown in Fig. 3. The areas around the plaque and carina had lower WSS. Stenting of bifurcation with plaque resulted in lower WSS than that of without plaque (0.46 Pa vs. 0.34 Pa) (Fig. 3). The simulations found that stenting of bifurcation with plaque resulted in higher stress ratio than that of without plaque (4.7 × 10^5^ vs. 7.9 × 10^5^) (Fig. 3C).

**Figure 4:**
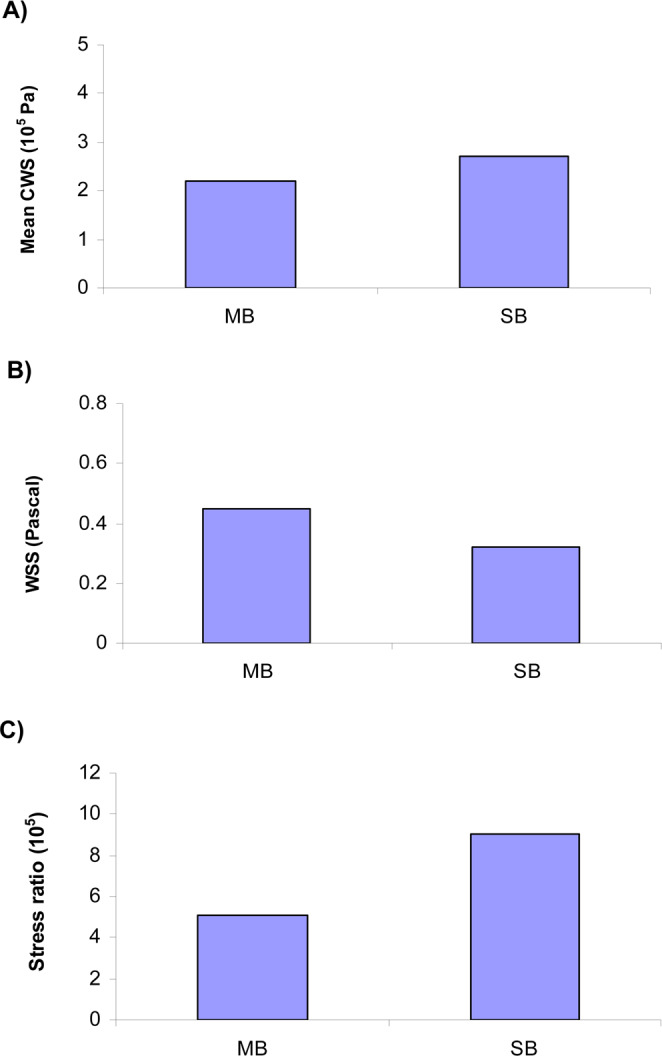
(**A**) Stenting of bifurcation with plaque resulted in higher CWS near the carina in SB than MB. (**B**) Stenting of bifurcation with plaque resulted in lower WSS in SB than MB. (**C**) The simulations predict the SB to have a nearly twice as large stress ratio as the MB (5.1 × 10^5^ vs. 9.2 × 10^5^).

**Figure 5:**
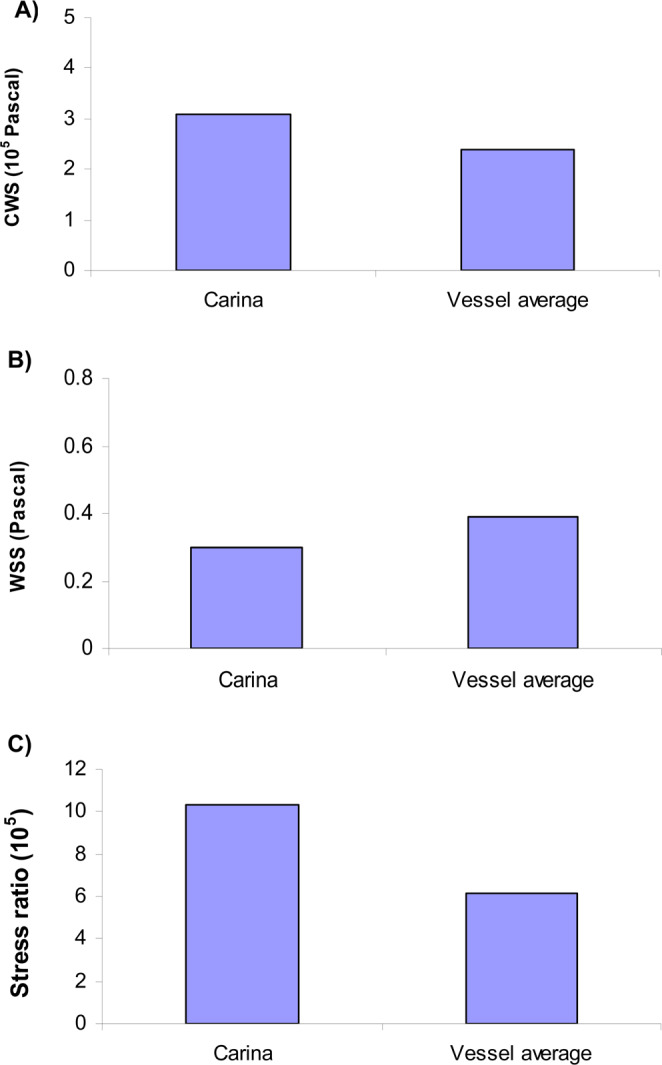
The simulations predict that the carina to have a much larger stress ratio as the vessel average, due to higher CWS and lower WSS at carina. The carina region is known to have higher incidences of stent thrombosis [Nakazawa *et al*., 2010].

Stenting of bifurcation with plaque resulted in higher mean CWS in SB than MB (290 KPa vs. 230 KPa) (Fig. 4A). Stenting of bifurcation resulted in lower WSS in SB than MB (0.47 Pa vs. 0.32 Pa) (Fig. 4B). The simulations predict the SB to have a nearly twice as large stress ratio as the MB (5.1 × 10^5^ vs. 9.2 × 10^5^) (Fig. 4C). The simulations further predicted that the carina has a much larger stress ratio than the vessel average, due to higher CWS and lower WSS (Fig. 5).

## Discussion

Treatment of coronary bifurcation lesions is still one of the most demanding procedures of interventional cardiology. The optimal technique for a particular bifurcation remains unclear due to the wide range of bifurcation anatomy. Numerical simulations is a powerful tool to model a variety of bifurcation geometries and quantify the effects of various stenting techniques on the vessel biomechanics. The major findings of this simulation study are that the decrease of WSS and increase in CWS are greater at SB than MB during provisional stenting. The results show the SB has a larger stress ratio than the MB. Higher re-stenosis rate at SB have been found clinically. The restenosis rate at SB was 14.7%, while much lower at 6.7% for the MB^4,7^. This is consistent with simulation findings that the SB to have a larger stress ratio than the MB (Fig. 4).

Intramural stresses are known to be stimuli for tissue remodeling, i.e., vessels develop hyperplasia to reduce intramural stresses. Additionally, low fluid WSS enhances inflammatory processes and promote thrombosis^10,12,14^. Higher restenosis rates of 15% and 28% in the SB were reported in clinical trials (CACTUS and Nordic bifurcation study) using DES^4,15^. In a study of patients with severe coronary disease, Nakazawa *et al*. found that late stent thrombosis was more prevalent near the carina region^16,17^. The simulations predicted that the carina has a much larger stress ratio than the center of plaque, due to higher CWS and lower WSS (Fig. 5).

It is worth noting that the SB ISR rate (15% to 28%)^4^) and thrombosis remain high for DES. The lack of hyperplasia on the struts of DES can cause persistent low fluid shear stress which may lead to stent thrombosis. Higher intramural stresses in the vessel wall are stimuli for inflammatory responses such as smooth muscle cell proliferation^10^. Additionally, the higher stresses can potentially cause stent and plaque fracture, leading to acute thrombosis. The frequency of late stent thrombosis was greater in the DES group (75%) than in the BMS group (36%)^16^.

### Limitations

This simulation study has the following limitations. First, the kissing-balloon technique was not simulated after provisional stenting of the MB. Kissing balloon is expected to open up the stenosis and struts in the SB near the carina, and normalize the WSS in SB. The simulation of strut interactions with plaque and balloon is beyond the scope of the current study. Second, the typical 60-degree angle was tested between the MB and the SB^18^. For the case of dedicated bifurcation stents, it may be helpful to evaluate the optimal angle as the angle can be adjusted in the device design. This issue may be investigated in future studies. Finally, for the outlet of the vessels, a traction free boundary condition was imposed in the current study. More severe stenosis (e.g., 90%) may affect the outlet traction significantly.

## Conclusions

The computational prediction of biomechanical stress ratios was consistent with clinical data on bifurcation stenting. Hemodynamics and solid mechanics are important factors to consider in planning of bifurcation stenting, as adverse biomechanics and hemodynamics may promote stent restenosis and thrombosis. These simulations may provide valuable insights for selecting optimal interventional strategy for various types of vascular bifurcations.

## Data Availability

All data generated or analyzed during this study are included in this manuscript.
